# Supplementary material for: Time-series transcriptome analysis identified differentially expressed genes in broiler chicken infected with mixed Eimeria species
Source: Front Genet. 2022 Aug 8;13:886781. doi: 10.3389/fgene.2022.886781 (PMC9393255; doi:10.3389/fgene.2022.886781)
Supplement: Supplementary file 2 [file DataSheet1.ZIP › 7dpi_KEGG.Gsea.1625115961950/KEGG_MTOR_SIGNALING_PATHWAY.html]

Details for gene set KEGG\_MTOR\_SIGNALING\_PATHWAY[GSEA]

|  || Dataset | TMM\_7dpi\_gct\_format\_7dpi\_gct\_format.Class\_4dpi.cls #PC\_versus\_NC.Class\_4dpi.cls #PC\_versus\_NC\_repos |
| Phenotype | Class\_4dpi.cls#PC\_versus\_NC\_repos |
| Upregulated in class | 0 |
| GeneSet | KEGG\_MTOR\_SIGNALING\_PATHWAY |
| Enrichment Score (ES) | -0.524348 |
| Normalized Enrichment Score (NES) | -1.7087557 |
| Nominal p-value | 0.0011330161 |
| FDR q-value | 0.1238691 |
| FWER p-Value | 0.2528 |
Table: GSEA Results Summary

  

Fig 1: Enrichment plot: KEGG\_MTOR\_SIGNALING\_PATHWAY      
 Profile of the Running ES Score & Positions of GeneSet Members on the Rank Ordered List

  

| SYMBOL | TITLE | RANK IN GENE LIST | RANK METRIC SCORE | RUNNING ES | CORE ENRICHMENT || 1 | ULK3 | na | 813 | 0.585 | -0.0453 | No |
| 2 | RPS6KB2 | na | 1071 | 0.495 | -0.0477 | No |
| 3 | PGF | na | 1105 | 0.483 | -0.0318 | No |
| 4 | EIF4EBP1 | na | 1169 | 0.467 | -0.0190 | No |
| 5 | CAB39L | na | 1494 | 0.382 | -0.0314 | No |
| 6 | EIF4B | na | 1912 | 0.286 | -0.0552 | No |
| 7 | RHEB | na | 2504 | 0.192 | -0.0972 | No |
| 8 | STK11 | na | 3068 | 0.117 | -0.1397 | No |
| 9 | CAB39 | na | 3390 | 0.075 | -0.1636 | No |
| 10 | RPS6KA1 | na | 3804 | 0.027 | -0.1971 | No |
| 11 | STRADA | na | 4503 | -0.050 | -0.2535 | No |
| 12 | MLST8 | na | 4597 | -0.060 | -0.2589 | No |
| 13 | EIF4E | na | 4735 | -0.074 | -0.2675 | No |
| 14 | VEGFC | na | 5000 | -0.103 | -0.2856 | No |
| 15 | HIF1A | na | 5064 | -0.109 | -0.2867 | No |
| 16 | EIF4E2 | na | 5204 | -0.122 | -0.2936 | No |
| 17 | RPTOR | na | 6743 | -0.279 | -0.4113 | No |
| 18 | AKT3 | na | 7033 | -0.312 | -0.4234 | No |
| 19 | PRKAA2 | na | 7484 | -0.360 | -0.4471 | No |
| 20 | RPS6 | na | 7644 | -0.376 | -0.4459 | No |
| 21 | PDPK1 | na | 8107 | -0.435 | -0.4677 | No |
| 22 | AKT1 | na | 8399 | -0.472 | -0.4738 | No |
| 23 | PIK3CG | na | 8478 | -0.481 | -0.4618 | No |
| 24 | VEGFA | na | 8816 | -0.526 | -0.4696 | No |
| 25 | VEGFD | na | 9472 | -0.627 | -0.5001 | Yes |
| 26 | RPS6KB1 | na | 9556 | -0.639 | -0.4824 | Yes |
| 27 | PIK3R2 | na | 9597 | -0.645 | -0.4609 | Yes |
| 28 | ULK2 | na | 9715 | -0.663 | -0.4450 | Yes |
| 29 | MTOR | na | 10269 | -0.763 | -0.4618 | Yes |
| 30 | MAPK3 | na | 10320 | -0.774 | -0.4361 | Yes |
| 31 | PIK3CB | na | 10335 | -0.777 | -0.4073 | Yes |
| 32 | TSC2 | na | 10538 | -0.818 | -0.3926 | Yes |
| 33 | RICTOR | na | 10686 | -0.851 | -0.3720 | Yes |
| 34 | PIK3CD | na | 10707 | -0.855 | -0.3406 | Yes |
| 35 | RPS6KA2 | na | 10791 | -0.876 | -0.3137 | Yes |
| 36 | PIK3CA | na | 10920 | -0.912 | -0.2892 | Yes |
| 37 | PIK3R5 | na | 10941 | -0.918 | -0.2555 | Yes |
| 38 | PIK3R3 | na | 11045 | -0.949 | -0.2274 | Yes |
| 39 | PIK3R1 | na | 11265 | -1.023 | -0.2062 | Yes |
| 40 | RPS6KA3 | na | 11288 | -1.031 | -0.1682 | Yes |
| 41 | ULK1 | na | 11308 | -1.040 | -0.1297 | Yes |
| 42 | PRKAA1 | na | 11327 | -1.049 | -0.0907 | Yes |
| 43 | RPS6KA6 | na | 11535 | -1.131 | -0.0643 | Yes |
| 44 | BRAF | na | 11724 | -1.264 | -0.0312 | Yes |
| 45 | IGF1 | na | 11859 | -1.426 | 0.0126 | Yes |
Table: GSEA details [plain text format]

  

Fig 2: KEGG\_MTOR\_SIGNALING\_PATHWAY      
 Blue-Pink O' Gram in the Space of the Analyzed GeneSet

  

Fig 3: KEGG\_MTOR\_SIGNALING\_PATHWAY: Random ES distribution      
 Gene set null distribution of ES for **KEGG\_MTOR\_SIGNALING\_PATHWAY**
